# Validation of novel low-dose CT methods for quantifying bone marrow in the appendicular skeleton of patients with multiple myeloma: initial results from the [^18^F]FDG PET/CT sub-study of the Phase 3 GMMG-HD7 Trial

**DOI:** 10.1007/s00259-025-07599-z

**Published:** 2025-10-01

**Authors:** Christos Sachpekidis, Marina Hajiyianni, Martin Grözinger, Maja Piller, Annette Kopp-Schneider, Elias K Mai, Lukas John, Sandra Sauer, Niels Weinhold, Ekaterina Menis, Olof Enqvist, Marc S. Raab, Anna Jauch, Lars Edenbrandt, Michael Hundemer, Alexander Brobeil, Johann Jende, Heinz-Peter Schlemmer, Stefan Delorme, Hartmut Goldschmidt, Antonia Dimitrakopoulou-Strauss

**Affiliations:** 1 Clinical Cooperation Unit Nuclear Medicine, German Cancer Research Center (DKFZ), Im Neuenheimer Feld 280, 69210 Heidelberg, Germany; 2 Heidelberg Myeloma Center, Internal Medicine V, Hematology, Oncology and Rheumatology, Heidelberg University Hospital, Heidelberg, Germany; 3 Division of Radiology, German Cancer Research Center (DKFZ), Heidelberg, Germany; 4 Medical Faculty Heidelberg, Heidelberg University, Heidelberg, Germany; 5 Division of Biostatistics, German Cancer Research Center (DKFZ), Heidelberg, Germany; 6 Eigenvision AB, Malmö, Sweden; 7 Department of Electrical Engineering, Chalmers University of Technology, Gothenburg, Sweden; 8 Institute of Human Genetics, University of Heidelberg, Heidelberg, Germany; 9 Department of Clinical Physiology, Region Västra Götaland, Sahlgrenska University Hospital, Gothenburg, Sweden; 10 Department of Molecular and Clinical Medicine, Institute of Medicine, Sahlgrenska Academy, University of Gothenburg, Gothenburg, Sweden; 11 Institute of Pathology, Heidelberg University Hospital, Heidelberg, Germany; 12 Internal Medicine V, Hematology, Oncology and Rheumatology, GMMG Study Group, Heidelberg University Hospital and National Center for Tumor Diseases, Heidelberg, Germany

**Keywords:** Multiple myeloma, [^18^F]FDG PET/CT, Whole-body low-dose CT, Induction therapy, Isatuximab, Minimal residual disease (MRD), Italian Myeloma criteria for PET Use (IMPeTUs), Artificial intelligence

## Abstract

**Purpose:**

The clinical significance of medullary abnormalities in the appendicular skeleton detected by computed tomography (CT) in patients with multiple myeloma (MM) remains incompletely elucidated. This study aims to validate novel low-dose CT-based methods for quantifying myeloma bone marrow (BM) volume in the appendicular skeleton of MM patients undergoing [^1^⁸F]FDG PET/CT.

**Materials and methods:**

Seventy-two newly diagnosed, transplantation eligible MM patients enrolled in the randomised phase 3 GMMG-HD7 trial underwent whole-body [^18^F]FDG PET/CT prior to treatment and after induction therapy with either isatuximab plus lenalidomide, bortezomib, and dexamethasone or lenalidomide, bortezomib, and dexamethasone alone. Two CT-based methods using the Medical Imaging Toolkit (MITK 2.4.0.0, Heidelberg, Germany) were used to quantify BM infiltration in the appendicular skeleton: (1) Manual approach, based on calculation of the highest mean CT value (CTv) within bony canals. (2) Semi-automated approach, based on summation of CT values across the appendicular skeleton to compute cumulative CT values (cCTv). PET/CT data were analyzed visually and via standardized uptake value (SUV) metrics, applying the Italian Myeloma criteria for PET Use (IMPeTUs). Additionally, an AI-based method was used to automatically derive whole-body metabolic tumor volume (MTV) and total lesion glycolysis (TLG) from PET scans. Post-induction, all patients were evaluated for minimal residual disease (MRD) using BM multiparametric flow cytometry. Correlation analyses were performed between imaging data and clinical, histopathological, and cytogenetic parameters, as well as treatment response. Statistical significance was defined as *p* < 0.05.

**Results:**

At baseline, the median CTv (manual) was 26.1 Hounsfield units (HU) and the median cCTv (semi-automated) was 5.5 HU. Both CT-based methods showed weak but significant correlations with disease burden indicators: CTv correlated with BM plasma cell infiltration (r = 0.29; *p* = 0.02) and β2-microglobulin levels (r = 0.28; *p* = 0.02), while cCTv correlated with BM plasma cell infiltration (r = 0.25; *p* = 0.04). Appendicular CT values further demonstrated significant associations with PET-derived parameters. Notably, SUVmax values from the BM of long bones were strongly correlated with both CTv (r = 0.61; *p* < 0.001) and moderately with cCTv (r = 0.45; *p* < 0.001). Patients classified as having increased [^1^⁸F]FDG uptake in the BM (Deauville Score ≥ 4), according to the IMPeTUs criteria, exhibited significantly higher CTv and cCTv values compared to those with Deauville Score <4 (*p* = 0.002 for both). AI-based analysis of PET data revealed additional weak-to-moderate significant associations, with MTV correlating with CTv (r = 0.32; *p* = 0.008) and cCTv (r = 0.45; *p* < 0.001), and TLG showing correlations with CTv (r = 0.36; *p* = 0.002) and cCTv (r = 0.46; *p* < 0.001). Following induction therapy, CT values decreased significantly from baseline (median CTv = -13.8 HU, median cCTv = 5.2 HU; *p* < 0.001 for both), and CTv significantly correlated with SUVmax values from the BM of long bones (r = 0.59; *p* < 0.001). In parallel, the incidence of follow-up pathological PET/CT scans, SUV values, Deauville Scores, and AI-derived MTV and TLG values showed a significant reduction after therapy (all *p* < 0.001). No significant differences in CTv, cCTv, or PET-derived metrics were observed between MRD-positive and MRD-negative patients.

**Conclusions:**

Novel CT-based quantification approaches for assessing BM involvement in the appendicular skeleton correlate with key clinical and PET parameters in MM. As low-dose, standardized techniques, they show promise for inclusion in MM imaging protocols, potentially enhancing assessment of disease extent and treatment response.

**Supplementary Information:**

The online version contains supplementary material available at 10.1007/s00259-025-07599-z.

## Introduction

Imaging plays a central role in the diagnosis and management of multiple myeloma (MM). Whole-body CT is the imaging modality of choice for detecting and assessing the extent of osteolytic lesions and is considered the minimum requirement at diagnosis, as per current guidelines [1]. [^18^F]FDG PET/CT is another high-performance imaging modality in MM management, considered the appropriate method for treatment response assessment [2–4]. Notably, according to the International Myeloma Working Group (IMWG), PET/CT may replace whole-body CT at initial diagnosis and is the preferred method in clinical trials for establishing a baseline for response assessment [1–6].

While identification of osteolytic lesions remains a key diagnostic criterion for MM and treatment initiation [5, 7], other imaging features, such as bone marrow (BM) abnormalities on low-dose CT, may provide additional insights. In healthy adults, the intramedullary cavities of the appendicular skeleton are predominantly occupied by fatty marrow [8]. In MM, however, plasma cell infiltration of these adipose-rich regions—most notably in the proximal femora and humeri—can be detected on CT as areas of increased attenuation. These abnormalities often precede cortical bone destruction and may present in diffuse or focal patterns [9]. Nevertheless, the clinical significance of these medullary findings remains poorly defined and they are not currently incorporated into the diagnostic criteria for myeloma bone disease.

The GMMG-HD7 trial is an ongoing, randomized, phase 3 study (NCT03617731) investigating the addition of isatuximab—a monoclonal anti-CD38 IgG1 antibody—to the standard induction regimen of lenalidomide, bortezomib, and dexamethasone (RVd) in newly diagnosed, transplant-eligible MM patients. Recently published results from part 1 of the trial showed that this quadruplet regimen significantly improved minimal residual disease (MRD) negativity rates and progression-free survival (PFS), suggesting a new standard of care in this setting [10, 11].

Here, we report findings from the imaging substudy of part 1 of the GMMG-HD7 trial. Specifically, we aimed to validate novel, low-dose CT-based techniques—acquired as part of [^1^⁸F]FDG PET/CT scans—for quantifying BM involvement in the appendicular skeleton before and after induction therapy in newly diagnosed, transplant-eligible MM patients.

## Materials and methods

### Patients

Between 23 October 2018 and 22 September 2020, 662 patients with newly diagnosed MM requiring systemic therapy, as defined by IMWG criteria [5], and eligible for high-dose melphalan, autologous stem cell transplantation (ASCT), and maintenance therapy, were enrolled in the GMMG-HD7 trial (NCT03617731) [10].

In part 1 of the study, all patients received up to three 42-day cycles of induction therapy. Both arms (isatuximab and control) received: lenalidomide (25 mg orally on days 1–14 and 22–35), bortezomib (1-3 mg/m^2^ subcutaneously on days 1, 4, 8, 11, 22, 25, 29, and 32) and dexamethasone (20 mg orally on days 1–2, 4–5, 8–9, 11–12, 15, 22–23, 25–26, 29–30, and 32–33). Patients in the isatuximab arm additionally received isatuximab (10 mg/kg intravenously on days 1, 8, 15, 22, and 29 of cycle 1, and on days 1, 15, and 29 of cycles 2 and 3). The primary endpoint of part 1 was the MRD-negativity rate (independent of IMWG response status) after induction therapy in the intention-to-treat population.

A subset of 72 patients (49 male, 23 female; median age 59 years) underwent [^1^⁸F]FDG PET/CT before and after induction therapy and were included in the PET/CT imaging substudy (Table 1). MRD status was assessed using bone marrow (BM) multiparametric flow cytometry. All patients gave written informed consent.

**Table 1:** Baseline patient characteristics (N = 72)

Patient characteristics	Value
Median age, years	59 (41 – 70)
Treatment group	
Isatuximab	33 (46%)
Control	39 (54%)
Sex	
Male	49 (68%)
Female	23 (32%)
Median haemoglobin, g/dL	11.7 (6.0 – 15.9)
Median albumin, g/dL	40.4 (21.2 – 54.3)
Median β2-microglobulin, mg/L	2.8 (0.9 – 16.3)
Median free light chain ratio (κ/λ)	12.0 (0.1 – 10546.9)
Median bone marrow plasma cell infiltration	55% (5 – 100%)
LDH levels	
pathologic	6 (8%)
normal	66 (92%)
High-risk cytogenetics†	
Yes	19 (26%)
No	49 (68%)
Unknown	4 (6%)
ISS	
1	43 (60%)
2	13 (18%)
3	16 (22%)
R-ISS	
1	29 (40%)
2	31 (43%)
3	8 (11%)
Not defined	4 (6%)

^†^High-risk cytogenetics defined as the presence of at least one of the following mutations: del(17)(p13), t(4;14)(p16;q32), or t(14;16)(q32;q23)

The study was approved by the ethics committee of the University of Heidelberg (AFmu-412/2018) and the German Federal Agency for Radiation Protection (ZD 3-22464/2023-116-A), and was conducted in accordance with the Declaration of Helsinki and the International Conference on Harmonisation Good Clinical Practice guidelines.

### Imaging data acquisition

All patients underwent whole-body [^1^⁸F]FDG PET/CT imaging at baseline and after induction therapy. Scanning was performed 60 minutes after intravenous injection of 3 MBq/kg [^1^⁸F]FDG using a Biograph mCT S128 PET/CT system (Siemens Healthineers, Erlangen, Germany). The scan covered the region from skull to toes, with 2 minutes per bed position. Low-dose CT (120 kV, 30 eff mA) without contrast was used for attenuation correction and anatomical correlation. PET images were reconstructed using an ordered subset expectation maximization (OSEM) algorithm with two iterations and 21 subsets, including time-of-flight (TOF) technology. The image matrix was 400 × 400 pixels.

### Imaging data analysis

#### CT-based quantification of BM density

In healthy adults, BM of the appendicular skeleton is usually replaced by adipose tissue, with CT values (CTv) ranging from −200 to −30 Hounsfield units (HU). Myelomatous infiltration raises CT attenuation to between −30 and 120 HU, while cortical bone exceeds 120 HU [12–14].

Two approaches were used to quantify BM infiltration of the appendicular skeleton:

- Manual approach: A radiologist and nuclear medicine physician (CS, MG) placed circular regions of interest (ROIs) in areas of elevated BM density (humeri and femora). The size of the ROI was determined according to the size of the respective high-density area in the visualized BM [13]. The highest mean CTv from these ROIs was recorded per patient.
- Semi-automated approach: Using the Medical Imaging Toolkit (MITK 2.4.0.0, Heidelberg, Germany), 3D volumes of interest (VOIs) were defined to exclude cortical bone (HU >120) and epiphyses, while including the diaphysis and metaphysis. Cumulative CT values (cCTv) were computed for all voxels within selected regions using the formula:
- Abnormal BM value (HU) = cumulative voxel HU × total volume of MM (mm^3^) / total volume of BM cavity (mm^3^) [14].

#### [^18^F]FDG PET/CT data analysis

Images were reviewed using a dedicated imaging workstation and software (aycan Osirix^PRO^) by two experienced nuclear medicine physicians (CS, ADS). Foci with [^1^⁸F]FDG uptake above background and not attributable to benign processes were considered MM-positive, even in the absence of corresponding CT osteolysis [15]. Readers were blinded to the treatment assignment of the enrolled patients (isatuximab vs. control).

Semi-quantitative analyses were performed using a dedicated software (PMOD Technologies, Zurich, Switzerland) (http://www.pmod.com/files/download/v31/doc/pbas/4729.htm).

by placing VOIs calculating standardized uptake value (SUV) over:

- MM lesions with highest [^1^⁸F]FDG uptake
- BM in lower lumbar spine, iliac crest (contralateral iliac crest if biopsied), and long bones (humeri, femora).
- Reference organs (liver and mediastinum), as suggested by the respective literature [16].

PET/CT data were further interpreted using the Italian Myeloma criteria for PET Use (IMPeTUs), which include [16, 17]:

- BM metabolism in the lower lumbar spine, based on the 5-point Deauville score (DS): score 1, no uptake at all; score 2, ≤ mediastinal blood pool uptake; score 3, >mediastinal blood pool uptake, ≤ liver uptake; score 4, > liver uptake + 10%; score 5, >> liver uptake (twice)
- [^18^F]FDG uptake intensity of the hottest MM lesion (DS 1-5)
- Number of focal, [^18^F]FDG-avid medullary lesions (Fx): F1, no lesions; F2, 1 - 3 lesions; F3, 4 - 10 lesions; F4, >10 lesions
- Presence of hypermetabolic paramedullary disease (PMD)
- Presence of hypermetabolic extramedullary disease (EMD)
- Number of lytic lesions on CT (Lx): L1, no lesions; L2, 1 - 3 lesions; L3, 4 - 10 lesions; L4, >10 lesions
- Presence of at least one fracture on CT.

Automated PET/CT image segmentation and volumetric quantification was also performed using a previously published deep learning pipeline [18, 19]. Briefly, the applied AI-based methodology consists of the following three steps:

1. CT-based organ segmentation: A convolutional neural network was used to segment the skeleton, liver, and muscle [20]. To avoid the effect of intense physiological [^18^F]FDG uptake in the brain, the skull was excluded from skeletal evaluations.
2. Application of SUV threshold in the skeleton: The CT-based segmentation was mapped onto the SUV PET images, after which an SUV threshold (≥ liver SUVmedian) was applied to detect BM infiltration [18, 19]. All pixels with SUV values above or equal to the threshold were classified as positive for MM infiltration, with additional steps implemented to reduce spillover effects from adjacent tissues into the bone mask caused by the limited resolution of PET imaging.
3. Post-processing and subsequent calculation of metabolic tumor volume (MTV) and total lesion glycolysis (TLG): Using the resulting masks, the total whole-body MTV (ml) was calculated as the volume of segmented pathological uptake in each patient. Specifically, MTV represents the volume of myeloma lesions detected on PET/CT with SUV values exceeding the predefined threshold. TLG (g) was then derived as the product of the mean SUV and MTV for the segmented regions (TLG= SUVmean x MTV).

### Clinical parameters, BM plasma cell infiltration, fluorescence in situ hybridization and clinical response to induction therapy

BM aspirates and trephine biopsies were obtained within four weeks of PET/CT and prior to treatment initiation. Bone marrow trephines were analysed using hematoxylin-eosin stain, periodic acid–Schiff stain and Giemsa stain. The percentage of BM infiltration by plasma cells was assessed via light microscope. The infiltration rate represents the number of plasma cells in comparison to all nucleated cells in BM. The monoclonality of plasma cells was confirmed by immunohistochemical staining.

Cytogenetic analyses were performed on CD138-purified cells, with high-risk features defined as del (17)(p13), t(4;14)(p16;q32), or t(14;16)(q32;q23) (cut-off, ≥10% of cells) [10]. Risk stratification followed the Revised International Staging System (R-ISS):

- Stage I**:** ISS I + no high-risk cytogenetics + normal lactate dehydrogenase (LDH)
- Stage III**:** ISS III + high-risk cytogenetics or high LDH
- Stage II**:** All others [21].

Treatment response was assessed within 7 days post-induction, including MRD evaluation using multiparametric next-generation flow cytometry (Multiple Myeloma Minimal Residual Disease Panel, EuroFlow, Cytognos S L, Salamanca, Spain; sensitivity: 10⁻^5^) [22], and IMWG response criteria including near-complete response (nCR) [10].

### Statistical analysis

Depending on the variables tested, the following approaches were applied for correlation and association analysis: for the correlation between continuous variables Spearman rank correlation coefficient, between nominal and ordinal variables chi-square tests of association, and between continuous and nominal variables Kruskal–Wallis test or in the case of two-group comparisons equivalently Wilcoxon rank sum test. Agreement was analysed by Cohens’ kappa coefficient. p-values below 0.05 were considered statistically significant. Due to the descriptive nature of the study, p-values were not adjusted for multiplicity. Calculations were performed with SAS 9.4.

## Results

### Patient cohort

#### Baseline

A total of 72 patients were included in the study, with 33 patients assigned to the isatuximab group and 39 to the control group. Plasma cell infiltration, assessed via BM biopsies and/or aspirates, ranged from 5% to 100%, with a mean of 50% and a median of 55%. Cytogenetic data were available for 68 patients (94%), revealing high-risk cytogenetic abnormalities in 19 of them (28%). Combined data on ISS and cytogenetics were also available for 68 patients. Based on this information, 29 patients (43%) were classified as R-ISS-1, 31 patients (46%) as R-ISS-2, and 8 patients (12%) as R-ISS-3. No significant differences in baseline characteristics were observed between the two treatment arms. Baseline patient characteristics are summarised in Table 1.

#### Follow-up after induction therapy

After induction therapy, MRD negativity was achieved in 18 patients (42%) in the isatuximab group and 14 patients (36%) in the control group (*p* = 0.11). According to IMWG response criteria, 30 patients (N = 10 isatuximab group, N = 20 control group) achieved CR or nCR. No differences in IMWG responses were observed between the two treatment arms. Response rates according to MRD status and IMWG criteria are summarised in Table 2.

**Table 2:** Response rates of the two treatment groups after induction therapy according to MRD status and IMWG criteria. Values refer to number of patients

	Treatment arm	
	Isatuximab	Control
IMWG response status		
CR	6	11
nCR	4	9
VGPR	16	8
PR	5	10
MR	2	0
SD	0	0
PD	0	1
MRD status		
negative	18	14
positive	15	25

*IMWG* International Myeloma Working Group; *CR* complete response; *nCR* near complete response; *VGPR* very good partial response; *PR* partial response; *MR* minimal response; *SD* stable disease; *PD* progressive disease; *MRD* minimal residual disease

### Imaging findings

#### Baseline

##### CT-based quantification of BM density in the appendicular skeleton

The median baseline CTv of the appendicular skeleton derived from the manual approach was 26.1 HU (mean CTv = 23.9 HU). The median baseline cCTv derived from the semi-automated approach was 5.5 HU (mean cCTv = 5.5 HU) (Table 3). No significant differences in baseline CT parameters of the appendicular skeleton were observed between the two treatment arms.

**Table 3:** CTv and cCTv values based on quantification of BM density in the appendicular skeleton. At follow-up, CTv and cCTv decreased significantly from baseline

	Baseline PET/CT	Follow-up PET/CT	*p* value
Median CTv (range), HU	26.1 (-65.1 – 87.3)	-13.8 (-81.8 – 88.5)	< 0.001
Median cCTv (range), HU	5.5 (4.8 – 7.4)	5.2 (4.4 – 7.1)	< 0.001

*CTv* CT values; *cCTv* cumulative CT values; *HU* Hounsfield units

**Fig. 1:**
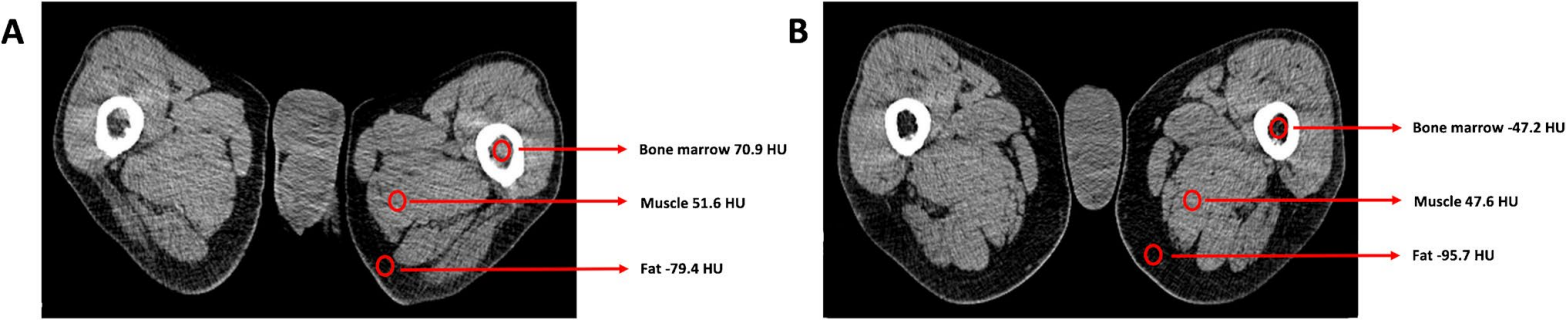
A previously untreated male MM patient with, R-ISS stage II, and a BM plasma cell infiltration rate of 76%. The patient received induction therapy with isatuximab in combination with lenalidomide, bortezomib, and dexamethasone. Following induction therapy, the patient achieved a very good partial response (VGPR) according to IMWG criteria, while MRD remained positive. Shown are representative axial CT images at the level of the femora and results from the manual CT-based quantification of BM infiltration in the appendicular skeleton before (**A**) and after (**B**) induction treatment. The highest mean BM CTv decreased from 32.7 HU to -26.6 HU, reflecting a treatment-associated reduction in BM density

**Fig. 2:**
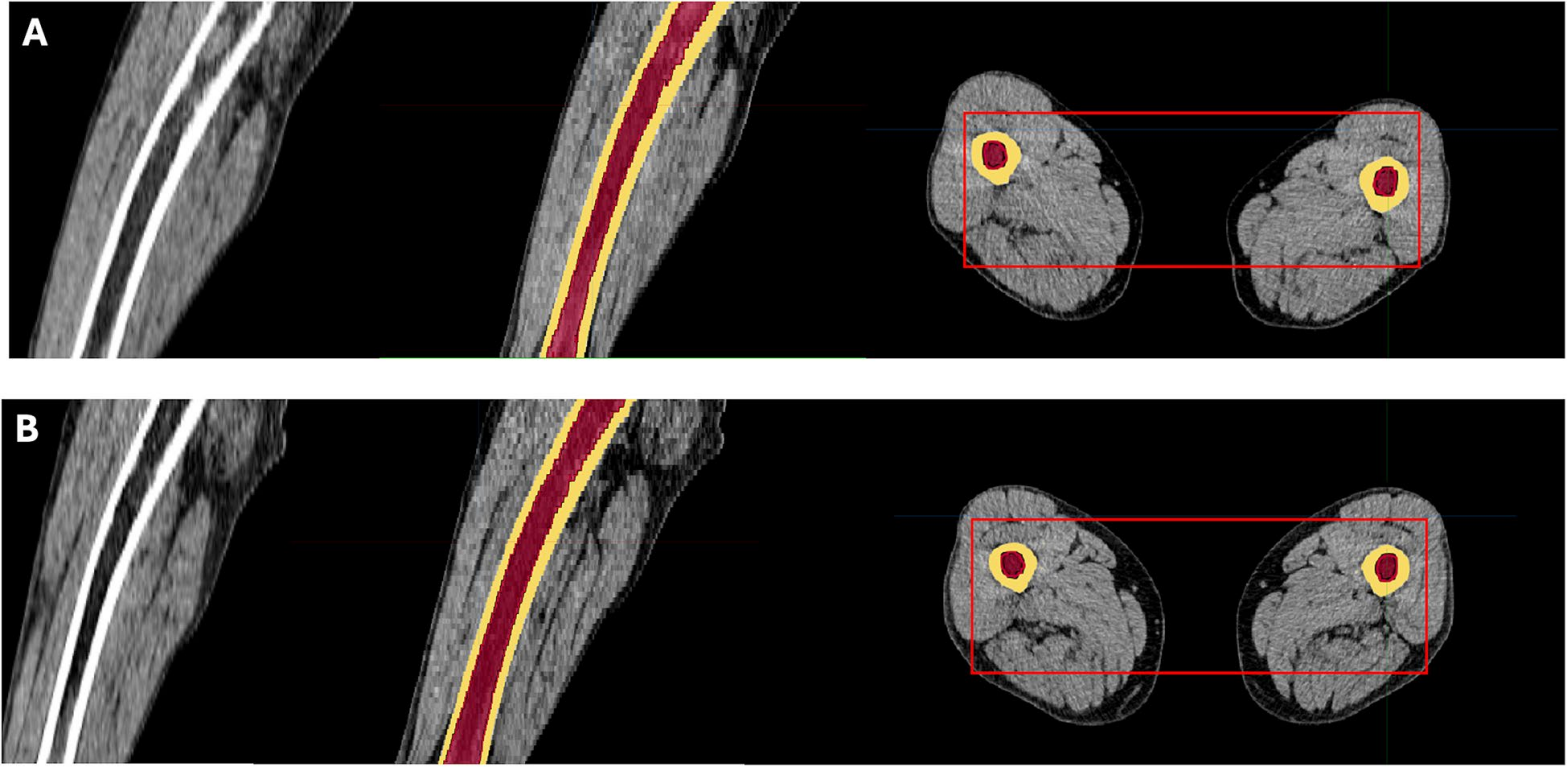
Representative images from the semi-automated CT-based quantification of BM infiltration in the appendicular skeleton for the same patient as in Fig. 1. Transaxial and sagittal CT images at the level of the femora are displayed. Three-dimensional volumes of interest (VOIs) were defined to exclude cortical bone (HU >120) and epiphyseal regions, while including the diaphysis and metaphysis. cCTv were calculated for all voxels within the selected regions. In this case, cCTv decreased from 6.70 HU at baseline (**A**) to 4.98 HU following induction treatment (**B**), indicating a reduction in BM density

At baseline, CT-based quantification of BM density in the appendicular skeleton showed a median manual CT value (CTv) of 26.1 HU, with a mean of 23.9 HU (Fig.1). The corresponding median semi-automated CT value (cCTv) was 5.5 HU, with a mean also of 5.5 HU (Fig. 2). No significant differences were observed between the treatment arms regarding baseline CT parameters of the appendicular skeleton.

##### PET/CT data analysis

**Fig. 3:**
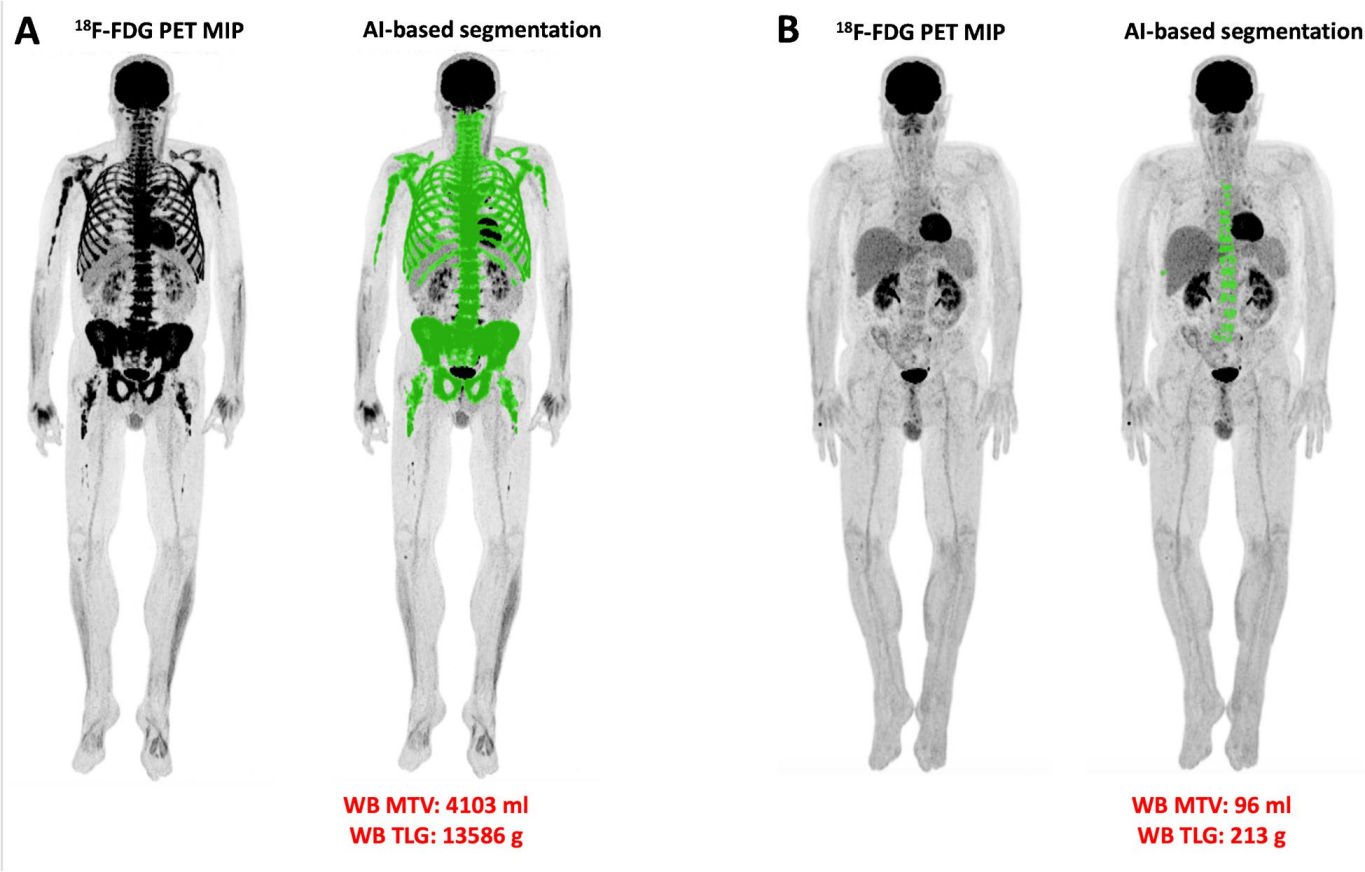
Representative images from the AI-based software tool applied to PET imaging, illustrating automated, volumetric quantification of BM metabolic activity in the same patient shown in Fig. 1. A marked reduction in whole-body MTV and TLG is observed from baseline (**A**) to follow-up after induction therapy (**B**)

Visual (qualitative) analysis of PET/CT scans showed that 58 of 72 patients (81%) had a pathological baseline PET/CT. According to the IMPeTUs criteria (Table 4), 37 patients (51%) exhibited a DS ≥4 for diffuse BM uptake, while 35 patients (49%) had DS <4. For the hottest focal lesions, 45 patients (63%) had DS ≥4, and 27 patients (37%) had DS <4. Focal medullary hypermetabolic lesions were not visually detectable (F1 score) in 26 patients (36%), while 46 patients (64%) presented at least one focal hypermetabolic lesion (median F score = 2). PMD and EMD were present in 23/72 (32%) and 3/72 (4%) patients, respectively. Eighteen patients (25%) had no osteolytic lesions (L1 score), whereas 54 patients (75%) showed at least one lytic lesion (median L score = 3). Fractures were detected in 43 patients (60%). The automated, AI-based PET/CT data quantification revealed a median whole-body MTV of 336 ml (mean MTV = 525 ml) and a TLG of 769 g (mean TLG = 1347 g) (Table 5) (Fig. 3). No significant differences in baseline PET/CT parameters were observed between the two treatment arms.

**Table 4:** PET/CT results after application of IMPeTUs

IMPeTUs criteria	Baseline PET/CT Patients (%)	Follow-up PET/CT Patients (%)
*Bone marrow uptake, DS*		
1	0	0
2	2 (2.8%)	16 (22.2%)
3	33 (45.8%)	53 (73.6%)
4	29 (40.3%)	2 (2.8%)
5	8 (11.1%)	1 (1.4%)
*No. of focal, hypermetabolic lesions*		
F_1_ (none)	26 (36.1%)	56 (77.8%)
F_2_ (1 - 3)	19 (26.4%)	12 (16.7%)
F_3_ (4 - 10)	13 (18.1%)	4 (5.6%)
F_4_ (>10)	14 (19.4%)	0
*Uptake of the hottest focal lesion, DS*		
1	26 (36.1%)	39 (54.2%)
2	0	13 (18.1%)
3	1 (1.3%)	6 (8.3%)
4	19 (26.4%)	9 (12.5%)
5	26 (36.1%)	5 (6.9%)
*No. of osteolyses*		
L_1_ (none)	18 (25.0%)	19 (26.4%)
L_2_ (1 - 3)	10 (13.8%)	8 (11.1%)
L_3_ (4 - 10)	8 (11.1%)	9 (12.5%)
L_4_ (>10)	36 (50.0%)	36 (50.0%)
*Presence of at least one fracture*		
no	29 (40.3%)	20 (27.8%)
yes	43 (59.7%)	52 (72.2%)
Presence of PMD*		
no	49 (68.1%)	69 (95.8%)
yes	23 (31.9%)	3 (4.2%)
Presence of EMD*		
no	69 (95.8%)	70 (97.2%)
yes	3 (4.2%)	2 (2.8%)

*IMPeTUs* Italian Myeloma criteria for PET Use; *DS* Deauville score; *PMD* paramedullary disease; *EMD* extramedullary disease

^*^Measurements refer to hypermetabolic lesions.

^§^Due to rounding, the % percentage values do not necessarily add to 100%

**Table 5:** MTV and TLG results after application of the AI-based tool for automated volumetric assessment of whole-body bone marrow metabolic activity. At follow-up, MTV and TLG decreased significantly from baseline

	Baseline PET/CT	Follow-up PET/CT	*p* value
Median MTV (range), mL	336 (7 – 4103)	102 (0 – 3294)	< 0.001
Median TLG (range), g	769 (22 – 13586)	227 (0 – 11114)	0.001

*MTV* metabolic tumor volume; *TLG* total lesion glycolysis

##### CT values of the appendicular skeleton correlate with clinical and PET parameters

Analysis of correlations showed that CTv was weakly but significantly associated with BM plasma cell infiltration (r = 0.29; *p* = 0.02) and serum β2-microglobulin levels (r = 0.28; *p* = 0.02). Similarly, cCTv correlated weakly but significantly with the plasma cell infiltration rate (r = 0.25; *p* = 0.04). A moderate-to-strong, positive significant correlation was observed between SUVmax from the BM of long bones and both CTv (r = 0.61; *p* < 0.001) and cCTv (r = 0.45; *p* = 0.001). SUV values of the reference iliac BM were also significantly associated with CTv (SUVmean: r = 0.30, *p* = 0.01; SUVmax: r = 0.30, *p* = 0.01) and cCTv (SUVmean: r = 0.35, *p* = 0.004; SUVmax: r = 0.31, *p* = 0.01).

Regarding IMPeTUs parameters, patients with a DS ≥4 for diffuse BM uptake had significantly higher CTv (DS≥4: mean CTv = 35.9; DS<4: mean CTv = 10.4) and cCTv values (DS≥4: mean cCTv = 5.7; DS<4: mean cCTv = 5.3) than those with DS <4 (*p* = 0.002 for both). By contrast, no significant correlations were found between quantitative CT values and the number of lytic lesions on CT (Lx). Likewise, CTv and cCTv did not differ significantly when patients were stratified by PMD or EMD status or by the presence of fractures.

Finally, AI-based PET/CT analysis revealed weak-to-moderate, significant positive correlations between CTv and cCTv with whole-body MTV (CTv: r = 0.32, *p* = 0.008; cCTv: r = 0.45, *p* < 0.001) and TLG (CTv: r = 0.36, *p* = 0.002; cCTv: r = 0.46, *p* < 0.001).

#### Follow-up after induction therapy

##### CT-based quantification of BM density in the appendicular skeleton

Following induction therapy, the median CTv of the appendicular skeleton significantly decreased from 26.1 HU (mean CTv = 23.9 HU) to -13.8 HU (mean = -5.7 HU) (*p* < 0.001) (Fig. 1). Similarly, the median cCTv significantly decreased from 5.5 HU (mean cCTv = 5.5 HU) to 5.2 HU (mean cCT= 5.3 HU) (*p* < 0.001) (Table 3) (Fig. 2). CTv significantly correlated with SUVmax values from the BM of long bones (r = 0.59; *p* < 0.001). No significant differences in CT parameters were observed between the two treatment arms after induction therapy.

##### PET/CT data analysis

PET/CT follow-up scans showed a significant reduction in pathological findings, with only 16 of 72 patients (22%) still displaying pathological scans compared to 81% at baseline (*p* < 0.001). SUV values from the BM of the lumbar spine, iliac bone and long bones, as well as the hottest focal MM lesions all significantly decreased from baseline (*p* < 0.001) (Suppl. Table 1). According to IMPeTUs criteria (Table 4), the incidence of patients with DS ≥4 for diffuse BM uptake significantly decreased from 51% to 4% (*p* < 0.001). Likewise, the incidence of DS ≥4 for the hottest focal lesions decreased from 63% to 19% (*p* < 0.001). Patients without detectable focal medullary lesions (F1 score) increased from 38% at baseline to 78% after therapy (*p* < 0.001). PMD positivity decreased markedly from 32% to 4% (*p* < 0.001), whereas EMD positivity remained stable (4% at baseline vs. 3% at follow-up). The number of osteolytic lesions (Lx score) did not significantly change, while the incidence of fractures increased (*p* = 0.004). AI-derived whole-body MTV and TLG showed significant reductions, with median MTV decreasing from 336 mL to 102 mL (*p* < 0.001) and median TLG from 769 g to 227 g (*p* = 0.001) (Fig. 3). No significant differences in PET-derived metrics were noted between the treatment arms. In addition, no significant differences were observed between MRD-positive and MRD-negative patients, nor across IMWG response groups, in any PET parameters, including the incidence of pathological PET/CT scans, SUV values, IMPeTUs parameters, or AI-derived MTV and TLG values.

##### CT values of the appendicular skeleton in relation to response to induction therapy

Consistent with PET parameters, no significant differences in either CTv or cCTv were observed between MRD-positive and MRD-negative patients. CT values also showed no significant variation according to response depth, as defined by the IMWG criteria. This held true whether comparing CR against all other response categories (nCR, very good partial response [VGPR], partial response [PR], minimal response [MR], stable disease [SD], and progressive disease [PD]) or combining CR and nCR versus the remaining response categories.

## Discussion

In this prospective imaging sub-analysis of the phase 3 GMMG-HD7 trial, we investigated novel CT-based approaches for assessing BM involvement in the appendicular skeleton of newly diagnosed, transplantation-eligible MM patients—both at baseline and following induction therapy. Our principal findings can be summarized as follows: First, low-dose CT-based segmentation and quantification of BM density in the appendicular skeleton proved technically feasible and yielded reliable measurements using both manual and semi-automated methods across all patients. Second, CT values derived from these methods showed weak-to-moderate but significant correlations with key clinical indicators of disease burden, including BM plasma cell infiltration rate, β2-microglobulin levels, as well as weak-to-strong significant correlations with metabolic tumor activity as measured by [^1^⁸F]FDG PET. Third, CT-based BM density values significantly decreased following induction therapy, indicating potential utility for monitoring treatment response.

Whole-body CT, either alone or in combination with PET, is the first technique recommended as a minimum requirement that should be used in MM, particularly for evaluating the extent of bone destruction [1]. In addition to its ability to depict lytic bone lesions - the imaging hallmark of MM - CT can also capture BM involvement in the appendicular skeleton, where hyperattenuating lesions often appear in adipocyte-rich tissue of long bones, usually preceding the destruction of mineralised bone [12, 23]. In an attempt to quantify the volume of myeloma infiltration in the BM of the appendicular skeleton, Nishida et al. developed methods to compute CT values of medullary density using both manual (CTv) and semi-automated (cCTv) techniques. Their work demonstrated associations between higher CTv and disease progression, with high cCTv emerging as an independent prognostic marker for overall survival [15, 16].

Our study is the first to externally validate these approaches in an independent patient cohort, further supporting their applicability in routine clinical and research settings. We observed a statistically significant, albeit weak, correlation between baseline CTv/cCTv and BM plasma cell infiltration rate from histology—a key disease biomarker [24]. In addition, CTv values correlated with β2-microglobulin, an established prognostic factor incorporated into ISS [25–27].

Importantly, the CT-derived metrics also correlated with various [^18^F]FDG PET-based parameters. For the first time, we demonstrate a moderate-to-strong positive correlation between both manual (CTv) and semi-automated (cCTv) measurements and SUVmax in the long bones, suggesting a link between BM density and metabolic activity in the appendicular skeleton. Whole-body MTV and TLG values—derived via a fully automated, AI-based method—also significantly correlated with CT-based BM infiltration. These PET-derived measures have been independently associated with prognosis in MM, further underscoring the clinical relevance of our findings [19]. Of note, although CTv and cCTv displayed largely similar correlation patterns with both clinical and PET parameters, some differences emerged. Specifically, only baseline CTv was significantly associated with β2-microglobulin levels (r = 0.28; *p* = 0.02), although cCTv showed a non-significant trend (r = 0.20; *p* = 0.09). At follow-up, CTv again correlated significantly with SUVmax in the BM of long bones (r = 0.59; *p* < 0.001), whereas cCTv demonstrated no association.

Using the IMPeTUs criteria—the first standardized framework for interpreting [^1^⁸F]FDG PET/CT in MM based on DS—we observed that patients exhibiting diffuse BM metabolism in the pelvic skeleton (DS ≥4) had significantly elevated CTv and cCTv values. This association was further supported by significant correlations between iliac BM [^1^⁸F]FDG uptake and CT-derived parameters. In newly diagnosed MM, higher BM DS values have previously been linked to greater disease burden and inferior outcomes [28, 29]. Our findings suggest that the corresponding increase in CT-based BM density in the appendicular skeleton may indicate more extensive plasma cell infiltration. These findings highlight the value of integrated PET/CT imaging—leveraging the functional insights of PET with the structural detail of CT—in enhancing disease characterization and refining risk stratification in newly diagnosed MM. Notably, CT-based assessment of the appendicular skeleton may serve as an additional imaging biomarker that could be incorporated into established interpretation frameworks such as the IMPeTUs criteria.

In the post-induction setting, CT-based BM density values decreased significantly compared to baseline, consistent with therapeutic response. This aligns with the high proportion of patients (96%) who achieved at least partial response per IMWG criteria. However, CT values did not vary significantly by depth of response–paralleling the PET results in our study. This mirrors previous findings by Nishida et al. [14] and suggests that while CT can track broad treatment effects, it may not discriminate between finer response categories in early treatment phases.

Similarly, BM density as assessed by CT was not associated with MRD status after induction therapy. This lack of association—also seen in PET-based assessments—may reflect the fact that follow-up focused on the post-induction phase, without accounting for further treatment such as HDT, ASCT, or maintenance. Within this context, the absence of a correlation may actually underscore the complementary nature of imaging and molecular techniques for residual disease monitoring in MM [30]. Longer-term follow-up data are needed to further evaluate this hypothesis.

The GMMG-HD7 trial is the first randomised phase 3 study to demonstrate improved induction therapy efficacy with the addition of isatuximab to standard treatment (lenalidomide, bortezomib, dexamethasone) in transplant-eligible MM patients. This was evidenced by higher MRD negativity rates and prolonged PFS in the isatuximab arm [10, 11]. However, this benefit in MRD status could not be confirmed in our imaging subcohort, likely due to the substantially smaller sample size (n = 660 in the full cohort vs. n = 72 in this analysis). In line with this, neither novel CT-based methods nor PET-derived metrics distinguished between treatment arms—an outcome not unexpected given limited statistical power. Additionally, limitations inherent to the imaging modalities used—such as the lower spatial resolution of low-dose CT and the nonspecific uptake of [^1^⁸F]FDG—may further reduce sensitivity in detecting subtle disease changes.

Our findings demonstrate that quantitative CT-derived parameters (CTv and cCTv) correlate weakly to moderately, yet significantly, with both clinical and PET-based measures in MM, indicating that they capture biologically relevant information. Nevertheless, the degree of overlap suggests that their incremental prognostic value over PET remains uncertain. Importantly, CT is more widely available and less costly than PET/CT, which may favor its clinical adoption, particularly in settings where PET/CT is not readily accessible. If future studies confirm that CT-derived metrics from the appendicular skeleton provide additive value beyond PET, their broader availability could make them a practical and cost-effective complement in routine MM response assessment. Due to the limited sample size of this subcohort, however, our study was not powered to establish independent prognostic significance of CT—using these evaluation approaches—as a stand-alone imaging modality. Larger prospective studies will therefore be required to clarify the role of CT-based measures relative to, and in combination with, PET.

In summary, [^1^⁸F]FDG PET/CT remains a valuable tool and is widely regarded as the most appropriate imaging modality for treatment monitoring in MM. However, its sensitivity appears lower than that of BM-based MRD assessment techniques, such as next-generation flow cytometry and next-generation sequencing, and it should therefore be applied as part of a broader multimodal assessment strategy [30–32].

We acknowledge some limitations of our study. First, the number of patients analyzed in this imaging substudy was small relative to the overall study population. Second, our findings are based on interim data limited to the post-induction phase; data from subsequent treatment phases (HDT, ASCT, maintenance) are forthcoming. As such, the current results should be interpreted as preliminary. Third, histopathologic confirmation of PET/CT findings was not feasible in most cases—a common challenge in clinical studies. To address this, we are conducting a separate investigation into the use of image-guided biopsies for molecular characterization of imaging-defined MM lesions [33].

## Conclusion

In this study, we evaluated novel low-dose CT-based techniques derived from whole-body [^18^F]FDG PET/CT imaging to quantify BM volume in the appendicular skeleton of newly diagnosed MM patients enrolled in the randomized phase 3 GMMG-HD7 trial. This trial investigates the addition of isatuximab to the standard induction regimen of lenalidomide, bortezomib, and dexamethasone. Our findings demonstrate the technical feasibility of BM density segmentation and quantification using both manual and semi-automated low-dose CT methods. The derived CT values correlated weakly to moderately—but significantly—with key clinical and metabolic [^18^F]FDG PET parameters, underscoring their potential as complementary imaging biomarkers to PET in MM. Importantly, these CT-based metrics also appear to be responsive to induction therapy, suggesting their applicability for early response assessment. However—similar to other PET-derived parameters—they did not distinguish patients based on MRD status at this stage of follow-up. The potential complementary role of hybrid PET/CT imaging—combining molecular insights from glucose metabolism with anatomical information for detecting lytic lesions and assessing BM infiltration—alongside flow cytometry for MRD monitoring, will be further explored in upcoming analyses incorporating long-term outcome data from the GMMG-HD7 trial.

## Supplementary Information

Below is the link to the electronic supplementary material.

Supplementary file1 (DOCX 14 KB)

## Data Availability

The datasets generated during and/or analysed during the current study are available from the corresponding author on reasonable request.
