# Supplementary material for: Validation of novel low-dose CT methods for quantifying bone marrow in the appendicular skeleton of patients with multiple myeloma: initial results from the [18F]FDG PET/CT sub-study of the Phase 3 GMMG-HD7 Trial
Source: Eur J Nucl Med Mol Imaging. 2025 Oct 1;53(3):1509–20. doi: 10.1007/s00259-025-07599-z (PMC12860749; doi:10.1007/s00259-025-07599-z)
Supplement: Supplementary file 1 — Supplementary file1 (DOCX 14 KB) [file 259_2025_7599_MOESM1_ESM.docx]

**Supplementary Table 1** SUV calculations of baseline and follow-up PET/CT studies. The calculations refer to median (range) values. At follow-up, all SUV values decreased significantly from baseline (p<0.05).

|  | **Baseline PET/CT** | **Follow-up PET/CT** |
| --- | --- | --- |
| **Parameter** |  |  |
| *BM* |  |  |
| SUV_mean_ lumbar spine | 2.44 (1.27-6.00) | 1.91 (0.65-5.68) |
| SUV_max_ lumbar spine | 3.29 (1.71-9.22) | 2.47 (0.96-8.38) |
| SUV_mean_ iliac bone | 2.16 (1.11-7.26) | 1.64 (0.71-7.23) |
| SUV_max_ iliac bone | 3.08 (1.62-11.06) | 2.19 (1.01-9.27) |
| *Hottest MM lesion* |  |  |
| SUV_mean_ | 4.76 (2.55-22.11) | 2.26 (1.07-6.26) |
| SUV_max_ | 6.65 (3.47-32.83) | 2.79 (1.50-7.34) |
| *Long bones* |  |  |
| SUV_mean_ BM of all extremities | 1.71 (1.04-2.22) | 1.64 (1.06-2.24) |
| SUV_max_ BM of all extremities | 2.13 (1.25-3.05) | 2.03 (1.41-2.79) |
| SUV_max_ hottest lesion of extremities | 2.43 (0.32-14.54) | 1.48 (0.30-5.50) |

BM, bone marrow; SUV, standardized uptake value; MM, multiple myeloma.
